# Uncovering Phenotypes in Sensorineural Hearing Loss: A Systematic Review of Unsupervised Machine Learning Approaches

**DOI:** 10.1097/AUD.0000000000001696

**Published:** 2025-08-07

**Authors:** Lilia Dimitrov, Liam Barrett, Aizaz Chaudhry, Jameel Muzaffar, Watjana Lilaonitkul, Nishchay Mehta

**Affiliations:** 1 University College London Hospital (UCLH) Biomedical Research Centre (BRC) Hearing Theme, London, United Kingdom; 2 University College London (UCL), London, United Kingdom; 3 University Hospitals Birmingham NHS Foundation Trust, Birmingham, United Kingdom.

**Keywords:** Artificial intelligence, Hearing loss, Machine learning, Phenotypes

## Abstract

**Objectives:**

The majority of the 1.5 billion people living with hearing loss are affected by sensorineural hearing loss (SNHL). Reliably categorizing these individuals into distinct subtypes remains a significant challenge, which is a critical step for developing tailored treatment approaches. Unsupervised machine learning, a branch of artificial intelligence (AI), offers a promising solution to this issue. However, no study has yet compared the outcomes of different AI models in this context. The purpose of this review is to synthesize the existing literature on the application of unsupervised machine learning models to hearing health data for identifying subtypes of SNHL.

**Design:**

A systematic search was performed of the following databases: MEDLINE, PsycINFO (Ovid version), EMBASE, CINAHL, IEEE, and Scopus as well as a search of grey literature using GitHub and Base, and manual search (Jan 1990–Mar 2024). Studies were included only if they reported on adult patients with SNHL and used an unsupervised machine-learning approach. Quality assessment was performed using the APPRAISE-AI tool. The heterogeneity of studies necessitated a narrative synthesis of the results.

**Results:**

Seven studies were included in the analysis. Apart from one case–control study, all were cohort studies. Four different algorithms were used, with no study comparing the performance of more than one algorithm. Across these studies, only 2 distinct numbers of subtypes were identified: 4 and 11. However, the overall quality of the studies was deemed low, thus preventing definitive conclusions regarding model selection and the actual number of subtypes.

**Conclusions:**

This systematic review identifies key methodological practices that need to be improved before the potential of unsupervised machine learning models to subtype SNHL can be realized. Future research in this field should justify model selection, ensure reproducibility, use high-quality hearing data, and validate model findings.

## INTRODUCTION

Through basic science research, our understanding of sensorineural hearing loss (SNHL) pathophysiology has increased, leading to the development of novel, targeted therapies (Schilder et al. 2019). However, despite nearly 20% of the world’s population having hearing loss (World Health Organization 2018), most of which is sensorineural, there are few disease-modifying treatment options actually available for patients. Translation from animal studies to clinical trials is hindered by the challenge of identifying patient subgroups (phenotypes) who may respond to these specialized treatments (Schilder et al. 2019, 2024).

Although our understanding of the pathology of SNHL has improved, we still lack the tools to identify the specific underlying causes of most SNHL cases in patients in vivo. Currently, reliable circulating biomarkers for this condition are lacking (Gomaa et al. 2020), and while underlying pathology in the most common causes of SNHL can be identified postmortem, this approach has limited practical application. As an alternative, pure tone audiometry (PTA), the most ubiquitous investigation performed for hearing loss, has been used to infer underlying pathology by suggesting that individuals with similar curve patterns share a common underlying cause (Schuknecht 1964; Schuknecht & Gacek 1993). This is supported by evidence, linking specific audiogram curve profiles to otological pathologies in both animal and human research (Schuknecht 1964; Schuknecht & Gacek 1993). This has led to the development of human-crafted classification systems rooted in these specific curve profiles (Schuknecht 1964; Schuknecht & Gacek 1993). However, recent advances in molecular and genetic techniques have challenged the underlying knowledge base underpinning these associations (Nelson & Hinojosa 2003). Furthermore, these systems only broadly distinguish between different causes of SNHL (Schuknecht 1964; Schuknecht & Gacek 1993).

To address some of these issues, there has been increasing focus on computational techniques to identify distinct groups of audiometric profiles, providing a more objective and data-driven approach to subtyping SNHL. In particular, unsupervised machine learning (UML), a branch of artificial intelligence (AI), has been successfully applied to disease phenotyping across a range of clinical specialties—including neurology (Eshaghi et al. 2021; Dadu et al. 2022), cardiology (Banerjee et al. 2023; Hu et al. 2024), and respiratory medicine (Angelini et al. 2023). In the context of SNHL, UML offers a promising approach for identifying subtypes—also referred to as clusters—by grouping individuals based on subtle patterns in hearing health data.

Unlike supervised machine learning (SML) methods, which benchmark success against a known ground truth, UML is particularly valuable in scenarios such as SNHL, where no such definitive reference exists. Although SML approaches have been applied using specific classification systems as “exemplars” or gold standards (Dubno et al. 2013), this inherently assumes the superiority of one among numerous pre-existing human-derived frameworks (Schmiedt 2010). UML approaches offer the potential to identify new, accurate classification systems for SNHL—whether by revealing subgroups not previously recognized, or by showing that traditionally separate categories in fact share a common underlying mechanism. Such UML methods underly several new proposed SNHL classification systems (Bisgaard et al. 2010; Cruickshanks et al. 2020).

Because UML relies on large volumes of data to detect patterns, its application in healthcare has grown alongside the increasing availability of electronic health records. Electronic health records provide the quantity and richness of real-world patient data needed to train and validate these complex models effectively at scale. Clustering methods used to identify disease phenotypes in this context are typically motivated by the potential to reveal subgroups that differ in clinically meaningful ways—such as prognosis, treatment response, or disease progression—and thus serve as a foundation for translation into clinical decision support. In the context of hearing health, a use case example could be patient stratification in clinical trials for emerging disease-modifying therapies by identifying individuals with the specific hearing loss etiology targeted by the intervention. In the longer term, such approaches would support the implementation of personalized medicine, ensuring that once new therapies are licensed, they can be matched to the patients most likely to benefit.

Using UML to identify disease subtypes is not without its own issues. These techniques are challenging to validate, and their outputs are highly dependent on the input data they are given (Basile & Ritchie 2018; Fleuren et al. 2020). Furthermore, interpreting the clusters identified by these models can be complex due to uncertain biological or clinical relevance, making it challenging to understand their relationship to disease mechanisms. Finally, many UML techniques exist, making it unclear which is optimal.

A systematic review of studies using UML techniques to subtype SNHL is needed to summarize current research including the clinical relevance and readiness of existing clustering techniques, highlight limitations, and guide future research directions in this rapidly growing field.

## MATERIALS AND METHODS

### Registration and Protocol

The review was registered in the PROSPERO database before starting the search (CRD42024532411). The initial review protocol and the subsequently amended protocol can be accessed on the PROSPERO website. The protocol was amended to improve the search strategy consistency across all databases after collaboration with a medical librarian.

### Study Design

This is a systematic review of UML studies using hearing health data to identify SNHL subtypes. It has been reported following the 2020 Preferred Reporting Items for Systematic Reviews and Meta-Analyses (PRISMA) guidelines.

### Eligibility Criteria

We included UML studies using hearing test data to identify SNHL subtypes in adults (aged ≥18 years). Children were excluded from this study because they have different underlying etiology to their hearing loss compared with an adult population and hearing test protocols and modalities differ based on age further limiting combining analysis with adults.

Only UML models are considered as we are interested in the generation of novel data-driven hearing loss subtypes rather than using pre-existing human-crafted classifications. The included studies should have hearing test data as the input to the UML model. This encompasses a wide range of investigations including PTA, EEG (including automated brainstem response), speech-in-noise testing, and otoacoustic emissions. We include English-language original research articles (see English-language section in Supplemental Digital Content 1, https://links.lww.com/EANDH/B677), conference proceedings as well as studies published on a popular repository used by software developers to encompass the wider outlets used by researchers within the machine learning field. The time period searched was between January 1, 1990, and April 24, 2024.

### Information Sources

The following databases were queried in this systematic review: Ovid EMBASE (from 1974), MEDLINE (Ovid), SCOPUS, IEEE Xplore, CINAHL Plus, PsycINFO (Ovid), GitHub Repository and BASE. Each database was last searched on May 15, 2024.

### Search Strategy

The search strategy was created in collaboration with a specialist medical librarian at University College London. A keyword search was performed common across all databases with the exception of GitHub where a tailored approach was used due to differences in repository structure and search functionality (see below). The keywords were selected to capture the concepts of hearing loss AND hearing tests AND machine learning. In addition to the keyword search, subject headings were also included in the search strategy tailored to each individual database. Initially, more specific keywords were applied but were later broadened to ensure the inclusion of key reference papers. This necessity, coupled with the large number of studies returned, highlights a limitation in current practices for title formulations, which often fail to precisely describe the type of AI application in relation to a specific clinical question. The keyword search strategy and full search strategy for each database can be found (Supplemental Digital Content 1, https://links.lww.com/EANDH/B677). For GitHub, where no clear title was given, the repository name was used.

**Fig. 1.**
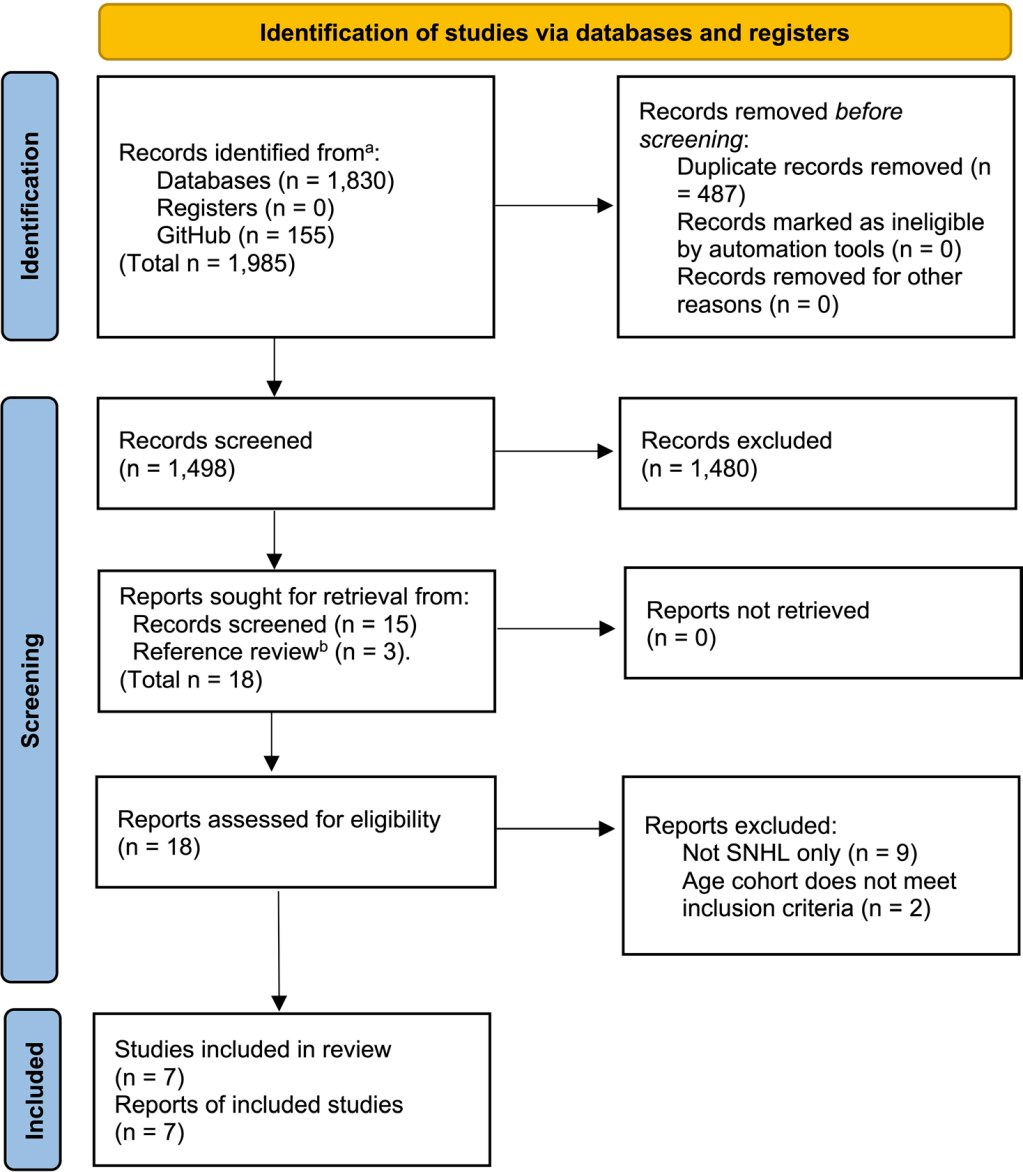
Flow diagram for screening process for the systematic review of UML approaches to phenotyping sensorineural hearing loss. ^a^A breakdown of records by database is available at the project webpage (see the availability of data, code, and other materials). ^b^Three additional papers were included via review of references during full-text review. SNHL indicates sensorineural hearing loss; UML, unsupervised machine learning.

### Selection Process

Two primary reviewers (L.B. and L.D.) conducted a two-stage screening process independently. In the first stage, titles and abstracts were screened based on the inclusion and exclusion criteria. In the second stage, full text was reviewed. The inter-rater reliability at the first stage was *k* = 0.82 and *k* = 0.88 at stage 2, indicating a near-perfect agreement at both stages (Landis & Koch 1977). For GitHub resources, where no abstract was given a suitable candidate was looked for in the project’s documentation or left null. The title and abstract were reviewed against the same inclusion/exclusion criteria above.

Of the 18 articles for full-text review, 7 were agreed to follow the necessary inclusion/exclusion criteria for inclusion in the current review. Any discrepancies between the two reviewers were resolved by discussion with a third author (N.M.).

### Data Collection Process

The same reviewers (L.D. and L.B.) independently extracted the data, with one reviewer (N.M.) resolving disagreements. The fields were manually entered into an Excel extraction template (Supplemental Digital Content 2, https://links.lww.com/EANDH/B678). The risk of bias assessment was performed using a distinct extraction template available from the APPRAISE-AI GitHub repository (Supplemental Digital Content 3, https://links.lww.com/EANDH/B679). Again, the same reviewers (L.D. and L.B.) independently extracted the data for this tool, with one reviewer (N.M.) resolving disagreements.

### Data Items

The primary co-outcomes are concerned with characterizing the SNHL subtypes identified by the machine learning algorithm. The measures of interest related to this outcome are (I) the number of clusters identified and (II) the characteristics (in terms of age, sex, and any other characteristics) of the identified clusters. The secondary co-outcomes relate to the validation of the identified SNHL subtypes. Validation of clustering studies is challenging given the lack of ground truth to generate measures of effect used in SML such as accuracy, sensitivity, recall, and so on. There are several alternatives to these measures to assess UML model validity such as predictive power of cluster membership to some other medical endpoint, measures of stability, and replicability when applied to additional datasets. Studies were assessed for whether (I) validation was performed (yes or no) and (II) the precise methods used. The primary and secondary outcomes were compared qualitatively across studies due to heterogeneity in the datasets and models.

In addition to the primary and secondary outcomes, other fields were extracted under the headings of study characteristics, dataset characteristics, dataset processing, model characteristics, validation, results, and discussion. The full list of fields is in Supplemental Digital Content 2, https://links.lww.com/EANDH/B678.

### Study Risk of Bias Assessment

APPRAISE-AI is a newly developed tool for quantitatively evaluating the quality of clinical AI studies across six domains: clinical relevance, data quality, methodological conduct, robustness of results, reporting quality, and reproducibility (Kwong et al. 2023). This tool consists of 24 items with a maximum score of 100 (Supplemental Digital Content 3, https://links.lww.com/EANDH/B679). The overall APPRAISE-AI score is graded such that scores of 0 to 19 indicate very low quality, 20 to 39 low quality, 40 to 59 moderate quality, 60 to 79 high quality, and 80 to 100 very high quality.

## RESULTS

The search strategy yielded 1985 texts of which 487 were duplicates and 1498 total unique results (Fig. 1). Fifteen articles were included for full-text review from the original results. An additional 3 papers were included from the examination of the references for the 15 articles. This resulted in a total of 18 articles for full-text review (Fig. 1). Seven of these articles were included in the study (Table 1). All included studies were cohort studies (five prospective and one retrospective), except for a single case–control study. The full details of the selection process are presented in the PRISMA flow diagram (Fig. 1). Of the 11 studies that were discarded from the 18, several initially appeared to meet the inclusion criteria but were excluded because the study cohorts were defined using air-conduction (AC) thresholds only therefore inadvertently including patients with conductive hearing loss (CHL) (Allen & Eddins 2010; Bisgaard et al. 2010; Cruickshanks et al. 2020; Elkhouly et al. 2022; Saak et al. 2022; Mamo et al. 2023; Anwar & Oakes 2012), and/or included children (Chang et al. 2019; Wahyudi et al. 2023). See Supplemental Digital Content 4, https://links.lww.com/EANDH/B680, for a breakdown of the excluded studies.

**TABLE 1.** Patient characteristics for each included study

Reference	Population	Participants Total (M, F)	Age Range (Mean, SD)	Hearing Loss Severity in dB HL (Mean, SD)	Type of Hearing Loss	Definition of SNHL
Lee et al. (2010)	Taiwan	1633 (M:719, F: 914)	M: 30–94, F: 30–91 (M: 59.8 ± 12.4, F: 58.0 ± 11.2)	M: 37 ± 23 F: 26 ± 19	Symmetrical SNHL	Excluded CHL but criteria not reported
Watanabe & Suzuki (2018)	Japan	115 (M: 56, F: 59)	11–84 (53.3)	Values not provided	Sudden SNHL	Criteria not reported
Parthasarathy et al. (2020)	USA	MEE: 132504 (M: 46–49%, F: 51–54%) NHANES: 15,340 (M/F: not reported)	MEE: 18–80 (63% of the sample >50 yr) NHANES: Not reported	Only figures displaying average audiograms per decade	SNHL	ABG <20 dB at one freq. or <15 dB at 2 consecutive freq.
Luo et al. (2013)	China	982 (M: 982, F: 0)	71–100 (80.84 ± 5.45)	44.02 ± 13.52	Symmetric SNHL	ABG ≤ 15 dB averaged across 0.5, 1, 2kHz
Wang et al. (2021)	China	Total: 10,307 (M: 88.9%, F: 10.1%) Dataset 1: 6631 (M: 88.3%, F: 11.7%) Dataset 2: 3676 (M: 90%, F: 10%)	Total: 16–60 (34.5 ± 8.8) Dataset 1: (36.2 ± 8.6) Dataset 2: (31.4 ± 8.3)	Values not provided	NIHL	Authors state NIHL is almost always SNHL, exclude abnormal tympanometry
Sanchez-Lopez et al. (2018)	Denmark, Spain	Spain: 68 (M: 43, F: 25) Denmark: 55 (M: 24, F: 31)	Spain: 25–82 (median 61) Denmark (HI): 52–80 (68.4) Denmark (NH): 41–70 (55.8)	Denmark: Low freq.: 24 ± 6, High freq.: 55 ± 6 Spain: Low freq.: 37 ± 12 dB, High freq.: 58 ± 12	SNHL and normal hearing	Spain: ABG ≤15 dB at 1 one freq. and ≤10 dB at any other freq. Denmark: excluded CHL but criteria not reported
Sanchez Lopez et al. (2020)	Denmark	75 (M: 37, F: 38)	59–82 (median 71)	Values not provided	Symmetrical SNHL and normal hearing	SNHL but criteria not reported

ABG, air-bone gap; F, female; freq, frequencies, HI, hearing impairment; M, male; MEE, Massachusetts Eye and Ear Hospital; NH, normal hearing; NHANES, National Health and Nutrition Examination Survey; NIHL, noise-induced hearing loss; SNHL, sensorineural hearing loss.

### Study Quality and Risk of Bias

**Fig. 2.**
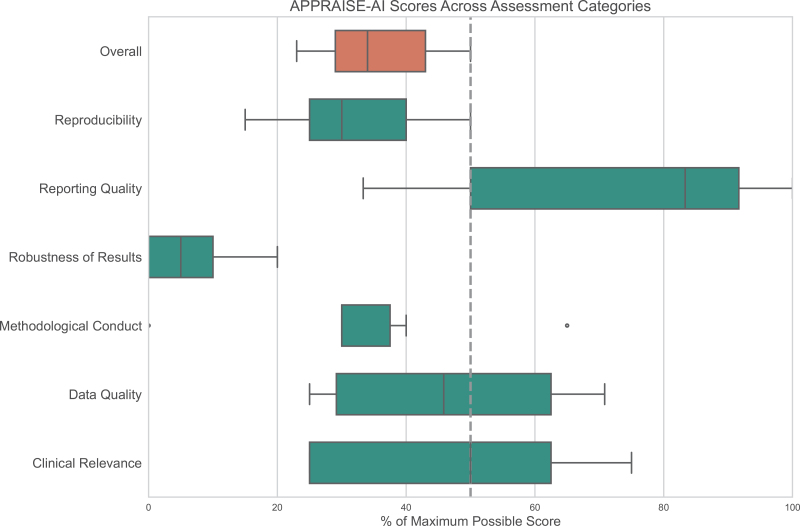
Assessment of APPRAISE-AI scores across evaluation domains. Box plots showing the APPRAISE-AI domain scores (teal) and overall score (coral) for the seven studies using UML to identify sensorineural hearing loss phenotypes. Scores are presented as percentages of the maximum possible score for each domain to enable comparison between categories with different weighting. The dashed vertical line at 50% provides a reference point. The quality assessment based on overall APPRAISE-AI scores ranged from low (20–39) to moderate (40–59), with most studies falling in the low-quality category.

The overall quality of each included study using the APPRAISE-AI tool is presented in Table 2 with the full results for each study in Supplemental Digital Content 5, https://links.lww.com/EANDH/B681. The overall and domain-specific scores across all included studies are presented visually in Figure 2. With the exception of two studies rated moderate (Luo et al. 2013; Wang et al. 2021), the majority of studies were of low quality (Lee et al. 2010; Sanchez Lopez et al. 2018, 2020; Watanabe & Suzuki 2018; Parthasarathy et al. 2020). Item 17 within the APPRAISE-AI Tool deals specifically with bias assessment. Barring one study (Wang et al. 2021), all studies score 0 out of a maximum possible score of 6 indicating a very high risk of bias. The studies scored poorly in this field because they did not evaluate model performance when stratified by patient-specific characteristics (such as age, gender, ethnicity) or by task-specific characteristics such as performance across different subtypes.

**TABLE 2.** Study design and UML model characteristics for each study included

Reference	Study Design	Variables	Clustering Method	Preprocessing	No. of Clusters	Cluster Characteristics	Validation of Clusters	Code/Data	Study Quality
Lee et al. (2010)	Retrospective cohort	PTA AC thresholds for 0.25, 0.5, 1, 2, 4, and 8 kHz	K means	All data adjusted by resetting 0.25 kHz to 0 dB, with all other frequencies shifted accordingly	11	Not reported except in terms of the hearing tests used in the model input	Not reported	Not reported	Low
Watanabe & Suzuki (2018)	Retrospective cohort	PTA AC thresholds but frequencies not specified	Hierarchical	Not performed	4	Duration of hearing loss pretreatment, tinnitus, vertigo, diabetes, nystagmus, canal paresis, mean hearing in unaffected ear	Internal validation using clinical outcome (cluster assignment to predict treatment response)	Not reported	Low
Parthasarathy et al. (2020)	Retrospective cohort	PTA AC thresholds for 0.5, 1, 2, 3, 4, 6, 8 kHz	GMM	Linear interpolation of 3 and 6 kHz in MEE dataset	NHANES: 6, MEE: 11	Age, sex, symmetry of hearing loss	External validation using additional dataset	On request	Low
Luo et al. (2013)	Case–control	PTA AC thresholds for 0.25, 0.5, 1, 2, 4, 6, 8 kHz.	K means	All data adjusted by resetting 0.25 kHz to 0 dB, with all other frequencies shifted accordingly	11 from cluster analysis of audiogram profile but reduced to 4 when genotype considered	Age, genotype	Internal validation using clinical outcome (cluster assignment and underling genotype)	Not reported	Moderate
Wang et al. (2021)	Prospective cohort	PTA AC thresholds for 0.5, 1, 2, 3, 4, 6, 8 kHz	K means	Not performed	4	Age, sex, race, job type, working time length, hearing protection use, earphone use, cigarettes, alcohol, noise exposure, hearing difficulty, tinnitus, BMI, noise exposure	Internal validation by running model on total dataset and on individual data subsets	On request	Moderate
Sanchez Lopez et al. (2018)	Retrospective cohort	A battery of hearing tests	Archetypal analysis	Data transformed so 25th percentile equals −0.5 and 75th percentile equals +0.5, dimensionality reduction with PCA	4	Not reported except in terms of the hearing tests used in the model input	Not reported	Not reported	Low
Sanchez-Lopez et al. (2020)	Retrospective cohort	A battery of hearing tests	Archetypal analysis	Data transformed so 25th percentile equals −0.5 and 75th percentile equals +0.5, dimensionality reduction with PCA	4	Not reported except in terms of the hearing tests used in the model input	Internal validation with bagging	Publically available	Low

GMM, Gaussian mixture model; MEE, Massachusetts Eye and Ear Hospital; NHANES, National Health and Nutrition Examination Survey; PCA, principal component analysis; PTA, pure tone audiometry; SNHL, sensorineural hearing loss.

### Study Population

Patient characteristics are summarized in Table 1. Sample sizes ranged widely from 55 to 132,504, with 3 studies including fewer than 125 patients (Sanchez Lopez et al. 2018, 2020; Watanabe & Suzuki 2018). Four studies included broadly equal numbers of men and women (Watanabe & Suzuki 2018; Parthasarathy et al. 2020; Sanchez-Lopez et al. 2020; Sanchez Lopez et al. 2018), two studies were either exclusively (Luo et al. 2013) or majority male (Wang et al. 2021), and only one study had a greater number of females (Lee et al. 2010). Participants were primarily from older age ranges. In almost all the studies, SNHL was defined using audiometric criteria although the exact definitions varied across studies (Lee et al. 2010; Luo et al. 2013; Sanchez Lopez et al. 2018, 2020; Watanabe & Suzuki 2018; Parthasarathy et al. 2020). One study used an inferred SNHL diagnosis derived from the presence of hearing loss using air-conduction thresholds without bone-conduction but the presence of normal tympanometry and occupational noise exposure (Wang et al. 2021). Most studies included patients with a general diagnosis of SNHL, one study looked at noise-induced hearing loss (Wang et al. 2021), another at age-related hearing impairment (Luo et al. 2013), and a third at sudden SNHL (Watanabe & Suzuki 2018).

### Evidence Synthesis of UML Models for SNHL

The results of individual studies are presented in Table 2. The results synthesis across these studies are explored under the subsequent subheadings.

#### UML Models

The UML algorithms assessed included k means (Lee et al. 2010; Luo et al. 2013; Wang et al. 2021), archetypal analysis (Sanchez Lopez et al. 2018, 2020), gaussian mixture model (Parthasarathy et al. 2020), and hierarchical analysis (Watanabe & Suzuki 2018). The justification of the choice of algorithm was not always reported (Lee et al. 2010; Wang et al. 2021), or concerningly, the only justification provided for the choice of algorithm was its previous usage in a different study without any specific reasons for its appropriateness (Luo et al. 2013). All included studies evaluated a single algorithm, with no study comparing the clustering solutions to different algorithms. The input to most of the models was PTA, except for two studies that used a large battery of tests (Sanchez Lopez et al. 2018, 2020).

#### Number of Clusters

The number of clusters identified ranged from 4 to 11 across all studies. Apart from hierarchical analysis (Watanabe & Suzuki 2018), all other models require identifying the number of clusters in advance. The techniques used to identify the optimal cluster number ranged being set a priori based on author’s hypothesis (Sanchez Lopez et al. 2018, 2020) to quantitative approaches seeking to optimize metrics (Lee et al. 2010; Luo et al. 2013; Parthasarathy et al. 2020; Wang et al. 2021). However, even in studies that used quantitative approaches for determining the optimal number of clusters, the techniques used included capping the maximum number of clusters without adequate explanation (Lee et al. 2010), suboptimal use of metrics (Parthasarathy et al. 2020; Wang et al. 2021) and disregard of the number of clusters identified by the UML model (Luo et al. 2013) .

#### Characteristics of Clusters

Characterization of the identified clusters in terms of demographic information and clinical characteristics varied across studies. Some studies purely described the clusters in terms of the hearing data used by the model (Lee et al. 2010; Sanchez Lopez et al. 2018, 2020). The majority of studies included information regarding sex and age, and some with additional fields such as hearing loss symptoms, genotype, comorbidities, and social behaviors (eg, smoking, alcohol, noise exposure) (Luo et al. 2013; Watanabe & Suzuki 2018; Parthasarathy et al. 2020; Wang et al. 2021). Where clusters were characterized by additional fields, only a minority contained any statistical analysis to see if these characteristics differed significantly between groups (Watanabe & Suzuki 2018; Wang et al. 2021) one of which did not describe which cluster showed a significant difference in the characteristic (although this is available on request to the author) (Watanabe & Suzuki 2018). One study only compared the clusters against healthy controls (Luo et al. 2013). This risks simply characterizing hearing loss versus non-hearing loss rather than the desired analysis: characterizing hearing loss subtypes.

#### Validation of Clusters

It is crucial to validate the identified subgroups from an UML as when given any dataset, UML models will inevitably identify clusters or groupings of data points. Two studies did not perform any validation of their clustering outcomes (Lee et al. 2010; Sanchez Lopez et al. 2018). Of the five studies that did perform validation, two studies performed external validation by comparing the clustering outcomes when their model was reapplied to multiple dataset (Parthasarathy et al. 2020; Wang et al. 2021). In the other three examples, two studies validated by using clinical measures to correlate with the clusters (Luo et al. 2013; Watanabe & Suzuki 2018), and one study, used a method called bootstrapping to re-run their model 1000 times using different forms of the original dataset (Sanchez-Lopez et al. 2020). Although it was promising that some form of validation was performed in the majority of studies, the quality of this validation was of a low standard and subject to methodological concerns, outlined in Table 3.

**TABLE 3.** Summary of key areas of weakness in the studies reviewed

Domain	APPRAISE-AI Score (Mean Average)	Specific Example	Issues Identified
Clinical Relevance	1.86/4	Choice of UML	No/limited justification given for model choice nor model limitations with respect to clinical considerations
Data Quality	11.14/24	Inclusion/exclusion criteria	Not always reported SNHL not identified appropriately, e.g., BC not measured
		Data preprocessing	Data transformed using poor/incorrect technique
Methodological Conduct	6.57/20	Baseline/comparator models	No attempt to compare model performance against alternative
		External validation/model generalizability	Results not validated using dataset from different institution Only qualitative analysis performed
		Correlation of disease-specific measures with clusters	No attempt to use clinical data to assess cluster validity e.g., hearing loss subtypes with underlying genotype
Robustness of Results	1.29/20	Internal validation	No/limited attempt to validate results using own dataset, e.g., bootstrapping, resampling, altering initialization
		Clinical utility assessment	Little to no attempt to test the clinical utility of the model
Reporting quality	8.57/12	Cluster characteristics	No/limited attempt to characterize identified clusters e.g. in terms of hearing severity, sex, age, etc.
Reproducibility	6.00/20	Data availability	Data not available
		Code availability	Code not available
		Model specification	Final hyperparameters not reported

BC, bone conduction; CHL, conductive hearing loss; UML, unsupervised machine learn.

## DISCUSSION

This is the first systematic review to assess the use of UML models to subtype SNHL. UML offers a framework for identifying hearing loss phenotypes and may, in time, contribute to improved understanding and treatment. However, we demonstrate that the current evidence base remains limited, and substantial methodological and data-related challenges must be addressed before any recommendations can be made on model choice or UML can reliably and safely support clinically meaningful subtyping of SNHL. This systematic review and risk of bias assessment identifies several key areas that future studies should address to improve research quality in this field. Specifically, the current evidence base is lacking in six critical domains: Clinical Relevance, Data Quality, Methodological Conduct, Robustness of Results, Reporting Quality, and Reproducibility (Table 3) which will be addressed in turn.

First, however, we will consider the primary outcome of this study which was to report the number of clusters identified across studies. Expert-derived systems have reported between four and seven SNHL subtypes (Carhart 1945; Schuknecht 1964; Schuknecht & Gacek 1993; Margolis & Saly 2007). We observed two recurring cluster solutions across the UML studies reviewed here: 4 and 11. The wide variation in the number of clusters identified across studies may reflect methodological inconsistencies and raises concerns about the robustness and biological relevance of the resulting groupings. However, the fact that multiple studies converged on two specific solutions suggests there may be some underlying structure in the data that these models are capturing. Although the majority of studies (4 of 7, see Supplemental Digital Content 2, https://links.lww.com/EANDH/B678) reported used optimization criteria to determine the number of clusters, these methods were often poorly described or inadequately justified. Conversely, nearly half of the studies did not explain their approach at all. This lack of transparency and consistency in cluster number selection likely contributes to the variation in the number of clusters identified across studies. One reason for the higher number of subtypes identified from some of the UML methods compared with traditional methods is that the traditional models have used audiometric configuration independently from hearing loss severity (i.e., the shape of the curve is the key component to the classification and not where the curve lies in terms of thresholds) (Schuknecht 1964; Schuknecht & Gacek 1993; Margolis & Saly 2007). Not considering severity at the same time as configuration could have a reductive effect in the number of subtypes.

A primary motivation for conducting this review is to support future researchers in selecting effective UML models from the vast number of available options. UML models are constrained by their underlying assumptions, and selecting an appropriate model requires careful consideration of how these assumptions align with the clinical problem being addressed (Alpaydin 2014). However, the majority of studies reviewed here did not adequately justify their choice of model or discuss its limitations within the specific study context.

A machine learning model’s effectiveness depends on the quality of the data it is trained on. The limited number of studies included in this review is partly due to studies opting to use only AC thresholds to define their hearing loss cohorts, resulting in cohorts where conductive and mixed hearing loss are unwittingly included. This is problematic as these conditions result from different sites of lesions. An additional important aspect of data quality in UML studies is the inclusion of relevant clinical measures to correlate with the identified clusters, as a means of assessing their meaningfulness in the absence of a ground truth. Although UML methods are adept at clustering data, it is imperative to assess the clinical relevance and utility of these groupings. Surprisingly, among the studies reviewed, only two of seven made efforts to link the identified clusters to clinical endpoints: one examined the response to steroid treatment in sudden SNHL (Watanabe & Suzuki 2018), while the other investigated the underlying genotype associated with the identified clusters (Luo et al. 2013). This highlights the necessity of validating UML groups against tangible clinical outcomes to ascertain their practical value and applicability in real-world settings.

In addition to data quality, sufficient data quantity is essential for reliable UML. Three of the studies included fewer than 125 patients, which is small for UML. Small datasets can lead to unstable or nonreproducible clusters due to an insufficient number of data points to clearly define them. Moreover, small datasets may fail to capture the full variability of the population, reducing the generalizability of the findings, and can also result in overfitting, where the model captures noise rather than meaningful patterns.

Studies also showed issues and limitations in terms of methodological conduct, particularly the assessment of model generalization which is especially critical in UML. Unlike supervised learning, where model output outcomes can be verified against known labels, UML models operate on unlabeled data, making validation more challenging. Generalization in this context refers to the model’s ability to identify meaningful and reproducible structure in unseen but comparable datasets. When demonstrated on independent datasets, this is often referred to as external validation. Replication across independent datasets strengthens the evidence that the observed clusters are not due to random variation, noise, or dataset-specific artifacts but reflect real, underlying biological phenomena. However, only two studies attempted external validation (Parthasarathy et al. 2020; Wang et al. 2021). In one case, this was problematic because the external dataset did not distinguish between conductive and SNHL, despite the internal dataset excluding CHL audiograms (Parthasarathy et al. 2020). This highlights the earlier concern about ensuring high data quality in clustering studies. The correlation of disease-specific measures with clusters can also support generalization, as biologically coherent clusters provide greater justification that the identified structure reflects real, generalizable patterns rather than artifacts of the specific dataset or method. As discussed earlier, this was only performed in two studies (Luo et al. 2013; Watanabe & Suzuki 2018).

A final aspect of methodological conduct relates to the lack of comparator models in these studies, which prevents an evaluation of how well the chosen model better captures or describes the underlying data structure relative to other approaches. Although we try to synthesize this data across different studies in this systematic review, lack of access to the data and heterogeneity across studies means this quantitative comparison cannot be performed. Notably, none of the included studies used a common dataset, limiting our ability to directly compare the outcomes of different clustering methods. Such an approach—applying multiple models to the same dataset and examining whether they converge on similar groupings—would provide valuable insight into whether the identified clusters reflect intrinsic patterns in the data or are artifacts of specific modeling choices.

Robustness refers to a model’s internal stability, in other words, whether it produces consistent clusters when faced with small perturbations either in the data or the model itself. There were examples where metrics for robustness were used to evaluate UML performance such as similarity of clusters across resampling but these represent a minority of studies (Sanchez-Lopez et al. 2020; Wang et al. 2021).

There was also often a lack of sufficient reporting of results. Phenotyping of SNHL aims to characterize underlying subtypes to guide treatment, prevent disease, and predict outcomes. However, many studies failed to adequately describe the identified audiometric subtypes in terms of patient characteristics. This lack of detailed reporting limits the understanding of the clinical implications and potential applications of the identified subtypes.

Finally, reproducibility was another area where studies underperformed. Inadequate model descriptions including failure to report final hyperparameter settings and/or unavailability of code/data were common observations, making it impossible to test reproducibility. Data availability is a significant challenge in health research due to the sensitive nature of health data and legal restrictions on data sharing. Solutions to address this include creation of publicly available datasets, generation of synthetic data (Chen et al. 2021), or use of novel techniques such as federated learning (Xu et al. 2021).

### Strengths and Limitations

In addition to being the first systematic review to tackle this topic, this study has several strengths. We used a robust and comprehensive search strategy to encompass the diversity of terms used to describe research in the machine learning field. Furthermore, we tailored our grey literature search strategy to the AI field by including GitHub.

In addition, bias was systematically assessed using a clinical research AI-specific tool: APPRAISE-AI. APPRAISE-AI remains the only validated tool specifically designed for the quantitative assessment of AI models in healthcare. Its structured scoring system enables direct comparisons across studies, which is particularly valuable in the context of a systematic review. The tool is however designed for use across a broad range of AI methodologies, including clustering. We acknowledge that not all the APPRAISE-AI domains are fully applicable to clustering models, potentially leading to lower scores and an underestimation of study quality. This raises the question of whether a single tool can effectively evaluate AI’s diverse methodologies or if adapted quality assessment tools are needed to address the unique characteristics of clustering algorithms and other AI approaches.

A core strength of APPRAISE-AI is that it was developed with clinical research in mind, specifically to evaluate AI models that support clinical decision-making. This emphasis is reflected in its structured scoring system. Although some of the studies included in this review have a narrower focus on knowledge discovery from clinical databases rather than direct clinical application, we suggest that separating clustering from clinical relevance in studies using patient data is an artificial distinction. Clustering methods used to identify disease phenotypes in this context are typically motivated by the potential to reveal subgroups that differ in meaningful ways—such as prognosis, treatment response, or disease progression—and thus serve as a foundation for clinical decision support.

Although knowledge discovery is a valuable scientific goal, clustering without any grounding in clinical or biological context is problematic. Algorithms will always generate groupings, but without appropriate validation, there is a substantial risk that these reflect statistical artifacts rather than biologically or clinically meaningful subtypes. For clustering to contribute meaningfully to clinical AI, it must be tied to the relevance of the identified groups—either directly through linkage to clinical endpoints or indirectly through robustness analyses, reproducibility, or external validation in independent datasets.

In this context, APPRAISE-AI is particularly valuable—not only for assessing methodological rigor but also for evaluating whether studies establish clinical relevance. This helps researchers and reviewers distinguish between exploratory analyses and findings with genuine translational potential. As such, its use represents an evidence-based best practice for evaluating the methodological quality and clinical relevance of AI research.

Aside from this, our search strategy yielded a large number of studies due to our use of broad search terms however this was necessary to capture landmark studies in the field which were missed when narrower terms were used. This highlights a limitation in current practices for academic paper title formulations in this field, which often fail to precisely describe the type of AI application in relation to a specific clinical question. Despite the high number of studies screened, only seven studies met the inclusion criteria. This may limit the scope of the review but reflects the methodological practices used in the available studies. Articles were predominantly excluded because they did not adequately distinguish between SNHL, CHL, or mixed patterns, by relying solely on AC thresholds as model input. These conditions reflect fundamentally different sites of lesion, and clustering without making this distinction is methodologically flawed, as it risks grouping together clinically and pathophysiologically distinct entities (Supplemental Digital Content 4, https://links.lww.com/EANDH/B680).

Even among the seven included studies, heterogeneous methodologies and outcomes pose a further challenge. As the number of AI models continues to grow, AI research is likely to become even more diverse. Our systematic review identifies recurrent poor practices that have persisted over the past 14 years and are common across the range of models in the current literature base. We believe that a systematic review of the literature is crucial at this time. By highlighting these issues and guiding readers to the APPRAISE-AI Toolkit, we aim to equip researchers with the knowledge to address these problems, thereby enhancing the quality of future research in this field. Finally, the grey literature search strategy could be extended to include other repositories such as Zenodo or OSF.

## CONCLUSIONS

AI, and UML in particular, has attracted growing interest as a potential tool for subtyping and understanding SNHL. However, our examination of the current body of evidence reveals significant shortcomings, with included studies demonstrating methodological weaknesses, a lack of reproducibility, and minimal validation. As a result, UML approaches for SNHL subtyping remain exploratory and substantial methodological refinement addressing these issues is needed before they can be confidently and safely applied in clinical or research contexts. In addition, efforts should be directed toward exploring methodologies to enhance research reproducibility, such as the development of purpose-built public hearing health datasets or the utilization of synthetic datasets.

## ACKNOWLEDGMENTS

The authors thank Heather Chesters for her invaluable assistance in performing the literature search.

## Supplementary Material
