# Supplementary material for: Uncovering Phenotypes in Sensorineural Hearing Loss: A Systematic Review of Unsupervised Machine Learning Approaches
Source: Ear Hear. 2025 Aug 7;46(6):1401–11. doi: 10.1097/AUD.0000000000001696 (PMC12533775; doi:10.1097/AUD.0000000000001696)
Supplement: Supplementary file 1 [file aud-46-1401-s001.pdf]

Supplemental Digital Content 1: Search Strategy

A keyword search was performed for each database. They keywords were selected to capture sensorineural hearing loss AND hearing tests AND machine learning. In addition to the keyword search, index terms were also included in the search strategy tailored to the database. The search strategy was evaluated by the confirmation of the presence of 5 key articles we would expect to be returned. The search terms below correspond to the particular database outlined.

Key words

(“Hearing loss” OR “Hearing Impair*” OR “sensorineural” OR “sensori neural” OR “age related hearing loss” OR presbyacusis or deaf*)
AND
(“pure tone audio*” OR audiometr* OR PTA OR “hearing test” OR “hearing assess*” OR “speech in noise” OR SIN OR “speech perception” OR ABR OR “auditory brainstem response” OR “auditory evoked potent*” OR AEP OR “otoacoustic emiss*” OR OAE)
AND
(“Machine learning” OR cluster* OR "artificial intelligence")

PUBMED:

Controlled Vocabulary
"Hearing loss"[MeSH Terms] AND (“Hearing tests”[MeSH Terms] OR "audiometry"[MeSH Terms] OR "otoacoustic emissions, spontaneous"[MeSH Terms] OR "evoked potentials, auditory, brain stem"[MeSH Terms] OR “speech perception”[MeSH Terms] OR “Auditory Threshold”[MeSH Terms]) AND ("cluster analysis"[MeSH Terms] OR "artificial intelligence"[MeSH Terms] OR “Neural Networks, Computer”[MeSH Terms] OR “Pattern Recognition, Automated” [MeSH Terms] OR “Data Mining”[MeSH Terms])

Full search strategy

(“Hearing loss” OR “Hearing Impair*” OR “sensorineural” OR “sensori neural” OR “age related hearing loss” OR presbyacusis OR deaf* **OR "Hearing loss"[MeSH Terms])** AND (**“Hearing tests”[MeSH Terms] OR "audiometry"[MeSH Terms]** OR “pure tone audio*” OR audiometr* OR auditory or **“Auditory Threshold”[MeSH Terms]** OR PTA OR “hearing test” OR “hearing assess*” OR “speech in noise” OR SIN OR “speech perception” OR **“speech perception”[MeSH Terms]** OR ABR OR “auditory brainstem response” OR “auditory evoked potent*” OR AEP OR “otoacoustic emiss*” OR OAE OR **"otoacoustic emissions, spontaneous"[MeSH Terms]** OR **"evoked potentials, auditory, brain stem"[MeSH Terms]**) AND (“Machine learning” OR cluster* OR “artificial intelligence” OR **"cluster analysis"[MeSH Terms]** OR **"artificial intelligence"[MeSH Terms]** OR **“Neural Networks, Computer”[MeSH Terms]** OR **“Pattern Recognition, Automated” [MeSH Terms]** OR **“Data Mining”[MeSH Terms]**)

Total: 878

SCOPUS:
Just keyword search as no subject headings used by this database. Keywords were searched in 'Article title, abstract, keywords'.

Full search strategy
("Hearing loss" OR "Hearing Impair*" OR "sensorineural" OR "sensori neural" OR "age related hearing loss" OR presbyacusis or deaf*) AND ("pure tone audio*" OR audiometr* OR auditory OR PTA OR "hearing test" OR "hearing assess*" OR "speech in noise" OR SIN or "speech perception" OR ABR OR "auditory brainstem response" OR "auditory evoked potent*" OR AEP OR "otoacoustic emiss*" OR OAE) AND ("Machine learning" or cluster* OR "artificial intelligence")

Total: 744

IEEE Xplore:
Just keyword search as no subject headings used by this database. Keywords were searched in 'All metadata'.

Full search strategy
("Hearing loss" OR "Hearing Impair*" OR "sensorineural" OR "sensori neural" OR "age related hearing loss" OR presbyacusis or deaf*) AND ("pure tone audio*" OR audiometr* OR auditory OR PTA OR "hearing test" OR "hearing assess*" OR "speech in noise" OR SIN or "speech perception" OR ABR OR "auditory brainstem response" OR "auditory evoked potent*" OR AEP OR "otoacoustic emiss*" OR OAE) AND ("Machine learning" or cluster* OR "artificial intelligence")

Total: 93

Ovid EMBASE (from 1974):

Controlled Vocabulary

(hearing impairment/ OR bilateral hearing loss/ OR cochlear synaptopathy/ OR functional hearing loss/ OR hereditary deafness/ OR congenital deafness/ OR high frequency hearing loss/ OR occupational deafness/ OR partial hearing loss/ OR perception deafness/ OR presbyacusis/ OR sudden deafness/ OR unilateral hearing loss/) AND
(pure tone audiometry/ OR hearing test/ OR speech perception/ OR auditory threshold/ OR auditory evoked potential/ OR evoked brain stem auditory response/) AND
(machine learning/ or cluster analysis/ OR exp artificial neural network/ OR automated pattern recognition/ OR exp data mining/ OR semi supervised machine learning/ or unsupervised machine learning/ OR exp artificial intelligence)

Full search strategy

(("Hearing loss" OR "Hearing Impair*" OR "sensorineural" OR "sensori neural" OR "age related hearing loss" OR presbyacusis or deaf*).ti,ab,kf. OR (**hearing impairment/** OR **bilateral hearing loss/** OR **cochlear synaptopathy/** OR **functional hearing loss/** OR **hereditary deafness/** OR **congenital deafness/** OR **high frequency hearing loss/** OR **occupational deafness/** OR **partial hearing loss/** OR **perception deafness/** OR **presbyacusis/** OR **sudden deafness/** OR **unilateral hearing loss/**)) AND (("pure tone audio*" OR audiometr* OR auditory OR PTA OR "hearing test" OR "hearing assess*" OR "speech in noise" OR SIN or "speech perception" OR ABR OR "auditory brainstem response" OR "auditory evoked potent*" OR AEP OR "otoacoustic emiss*" OR OAE).ti,ab,kf. OR (**pure tone audiometry/** OR **hearing test/** OR **speech perception/** OR **auditory threshold/** OR **auditory evoked potential/** OR **evoked brain stem auditory response/**)) AND (("Machine learning" or cluster* OR

"artificial intelligence").ti,ab,kf. OR (**machine learning/** OR cluster analysis/ OR **exp artificial neural network/** OR **automated pattern recognition/** OR **exp data mining/** OR **semi supervised machine learning/** or **unsupervised machine learning/** OR **exp artificial intelligence/**))

Total: 568

CINAHL Plus:

Controlled vocabulary

(MH “hearing disorders+” and (MH “Hearing tests” OR MH “Evoked Potentials, Auditory” OR MH “Audiometry, Pure Tone” OR MH “Audiometry, Evoked Response” OR MH “Otoacoustic emissions, spontaneous ” OR MH “Speech Perception” OR MH “Auditory Threshold”) AND MH "artificial intelligence" OR MH “Machine Learning” OR MH “Cluster Analysis” OR MH “Neural Networks (Computer)” OR MH “Data Mining”)

Full search strategy

No specific search fields were selected.

(**MH “Hearing Disorders+”** OR "Hearing loss" OR "Hearing Impair*" OR "sensorineural" OR "sensori neural" OR "age related hearing loss" OR presbyacusis or deaf*) AND (**MH “Hearing tests” OR MH “Evoked Potentials, Auditory”** OR **MH “Audiometry, Pure Tone”** OR **MH “Audiometry, Evoked Response”** OR **MH “Otoacoustic emissions, spontaneous ”** OR **MH “Speech Perception”** OR **MH “Auditory Threshold”** OR "pure tone audio*" OR audiometr* OR auditory OR PTA OR "hearing test" OR "hearing assess*" OR "speech in noise" OR SIN or "speech perception" OR ABR OR "auditory brainstem response" OR "auditory evoked potent*" OR AEP OR "otoacoustic emiss*" OR OAE) AND (**MH "Artifical Intelligence"** OR **MH “Machine Learning”** OR **MH “Cluster Analysis”** OR **MH “Neural Networks (Computer)”** OR **MH “Data Mining”** OR “Machine learning” or cluster* OR "artificial intelligence")

Total: 168

PsychINFO (Ovid):

Controlled Vocabulary

(hearing loss/ or hearing disorders/ or deafness/) and (audiometry/ or auditory thresholds/ or speech perception/ or auditory evoked potentials/) and (machine learning/ or machine learning algorithms/ or predictive analysis/ or unsupervised learning/ or "clustering (machine learning)"/ or data mining/ or artificial intelligence/ or artificial neural networks/)

Full search strategy

(**hearing loss/** or **hearing disorders/** or **deafness/** or ("Hearing loss" or "Hearing Impair*" or "sensorineural" or "sensori neural" or "age related hearing loss" or presbyacusis or deaf*).ti,ab,id.) and (**audiometry/** or **auditory thresholds/** or **speech perception/** or **auditory evoked potentials/** or ("pure tone audio*" or audiometr* or PTA or "hearing test" or "hearing assess*" or "speech in noise" or SIN or "speech perception" or ABR or "auditory brainstem response" or "auditory evoked potent*" or AEP or "otoacoustic emiss*" or OAE).ti,ab,id.) and (**machine learning/** or **machine**

learning algorithms/ or **predictive analysis/** or **unsupervised learning/** or **"clustering (machine learning)"/** or **data mining/ or artificial intelligence/** or **artificial neural networks/** or (“Machine learning” or cluster* or "artificial intelligence").ti,ab,id.)

Total: 58

BASE:

Note: base is a search engine and not a subject database. As such it does not support complex search strategies such as the keyword strategy. As such an adapted search was performed.

Final search strategy
"Hearing Loss AND Machine Learning"
"Hearing Loss AND Cluster Analysis"
"Hearing Loss AND Artificial Intelligence"
"Audiometr* AND Machine Learning"
"Audiometr* AND Cluster Analysis"
"Audiometr* AND Artificial Intelligence"

GitHub repository:
Github repository is not a subject database. As such it does not support complex search strategies such as the keyword strategy. As such an adapted search was performed.

Final search strategy
“audiometric created:2014 created:2015 created:2016 created:2017 created:2018 created:2019 created:2020 created:2021 created:2022 created:2023 created:2024-01 created:2024-02 created:2024-03"

“audiometry created:2014 created:2015 created:2016 created:2017 created:2018 created:2019 created:2020 created:2021 created:2022 created:2023 created:2024-01 created:2024-02 created:2024-03"

“audiology created:2014 created:2015 created:2016 created:2017 created:2018 created:2019 created:2020 created:2021 created:2022 created:2023 created:2024-01 created:2024-02 created:2024-03"

Total: 155

English-only

English-language studies were included as all authors are English speakers, and the technical nature of the papers made us hesitant to rely on AI translation software, which might not accurately convey their meaning. Additionally, hiring translators for potentially numerous languages was not a practical option. However, we did not explicitly restrict our search strategy to English-only studies as shown in the above search terms.

Upon post-hoc review of the raw search results, a python script was made to automate language detection. This identified 125 records. However on manual review only 7 of these records were non-English. None of these 7 articles met the inclusion/ exclusion criteria.
