# Supplementary material for: Uncovering Phenotypes in Sensorineural Hearing Loss: A Systematic Review of Unsupervised Machine Learning Approaches
Source: Ear Hear. 2025 Aug 7;46(6):1401–11. doi: 10.1097/AUD.0000000000001696 (PMC12533775; doi:10.1097/AUD.0000000000001696)
Supplement: Supplementary file 4 [file aud-46-1401-s004.pdf]

Supplemental Digital Content 4: Table of excluded studies

Study reference	Reason for exclusion
Elkhouly et al, 2022(Elkhouly et al., 2022)	Only considered AC values with no specific mention of interest in SNHL therefore may contain patients with CHL
Cruickshanks et al, 2020(Cruickshanks et al., 2020)	Cohort contained CHL and these patients were not identified in the cluster analysis
Anwar et al, 2012(Anwar & Oakes, 2012)	Cohort contains patients with ABG
Chang et al, 2019(Chang et al., 2019)	Only considered AC values with no specific mention of interest in SNHL therefore may contain patients with CHL<br>Includes children (>12 years)
Wahyudi et al, 2023	Minimal information given about testing procedure<br>No inclusion criteria given<br>No mention of SNHL in the article<br>May include children - high school students used
Saak et al, 2022(Saak et al., 2022)	Cohort contained CHL and these patients were not identified in the cluster analysis
Allen et al, 2010(Allen & Eddins, 2010)	Only considered AC values with no specific mention of interest in SNHL therefore may contain patients with CHL
Cilleti et al, 2008(Ciletti & Flamme, 2008)	Only considered AC values with no specific mention of interest in SNHL therefore may contain patients with CHL
Mamo et al, 2010(Mamo et al., 2023)	Only considered AC values with no specific mention of interest in SNHL therefore may contain patients with CHL<br>Aim is not to find clusters of hearing loss subtypes
Belitz et al, 2019(Belitz et al., 2019)	Only considered AC values with no specific mention of interest in SNHL therefore may contain patients with CHL<br>Clusters based on HA setting rather than a measure of hearing loss
Bisgaard et al, 2010(Bisgaard et al., 2010)	Only considered AC values with no specific mention of interest in SNHL therefore may contain patients with CHL

Abbreviations: ABG, air-bone gap; AC, air conduction; CHL, conductive hearing loss; HA, hearing aid; SNHL, sensorineural hearing loss

Allen, P. D., & Eddins, D. A. (2010). Presbycusis phenotypes form a heterogeneous continuum when ordered by degree and configuration of hearing loss. *Hearing Research*, *264*(1), 10-20. https://doi.org/https://doi.org/10.1016/j.heares.2010.02.001

Anwar, M. N., & Oakes, M. P. (2012). Data mining of audiology patient records: factors influencing the choice of hearing aid type. *BMC Med Inform Decis Mak*, *12 Suppl 1*(Suppl 1), S6. https://doi.org/10.1186/1472-6947-12-s1-s6

Belitz, C., Ali, H., & Hansen, J. H. L. (2019). A Machine Learning Based Clustering Protocol for Determining Hearing Aid Initial Configurations from Pure-Tone Audiograms. *Interspeech*, *2019*, 2325-2329. https://doi.org/10.21437/interspeech.2019-3091

Bisgaard, N., Vlaming, M. S., & Dahlquist, M. (2010). Standard audiograms for the IEC 60118-15 measurement procedure. *Trends Amplif*, *14*(2), 113-120. https://doi.org/10.1177/1084713810379609

Chang, Y. S., Yoon, S. H., Kim, J. R., Baek, S. Y., Cho, Y. S., Hong, S. H., Kim, S., & Moon, I. J. (2019). Standard Audiograms for Koreans Derived through Hierarchical Clustering Using Data from the Korean National Health and Nutrition Examination Survey 2009-2012. *Sci Rep*, *9*(1), 3675. https://doi.org/10.1038/s41598-019-40300-7

Ciletti, L., & Flamme, G. A. (2008). Prevalence of hearing impairment by gender and audiometric configuration: results from the National Health and Nutrition Examination Survey (1999-2004) and the Keokuk County Rural Health Study (1994-1998). *J Am Acad Audiol*, *19*(9), 672-685. https://doi.org/10.3766/jaaa.19.9.3

Cruickshanks, K. J., Nondahl, D. M., Fischer, M. E., Schubert, C. R., & Tweed, T. S. (2020). A Novel Method for Classifying Hearing Impairment in Epidemiological Studies of Aging: The Wisconsin Age-Related Hearing Impairment Classification Scale. *Am J Audiol*, *29*(1), 59-67. https://doi.org/10.1044/2019_aja-19-00021

Elkhouly, A., Andrew, A. M., Rahim, H. A., Abdulaziz, N., Abdulmalek, M., Mohd Yasin, M. N., Jusoh, M., Sabapathy, T., & Siddique, S. (2022). A Novel Unsupervised Spectral Clustering for Pure-Tone Audiograms towards Hearing Aid Filter Bank Design and Initial Configurations. *Applied Sciences*, *12*(1).

Mamo, S. K., Pearlman, J., & Wheeler, K. A. (2023). Associations Between Age-Related Hearing Loss, Cognitive Impairment, and Multiple Chronic Conditions in a Group Care Setting. *J Speech Lang Hear Res*, *66*(12), 5087-5108. https://doi.org/10.1044/2023_jslhr-23-00067

Saak, S., Huelsmeier, D., Kollmeier, B., & Buhl, M. (2022). A flexible data-driven audiological patient stratification method for deriving auditory profiles. *Front Neurol*, *13*, 959582. https://doi.org/10.3389/fneur.2022.959582
